# Identification of (poly)phenol treatments that modulate the release of pro-inflammatory cytokines by human lymphocytes

**DOI:** 10.1017/S0007114516000805

**Published:** 2016-03-17

**Authors:** Christopher T. Ford, Siân Richardson, Francis McArdle, Silvina B. Lotito, Alan Crozier, Anne McArdle, Malcolm J. Jackson

**Affiliations:** 1 Department of Musculoskeletal Biology, Institute of Ageing and Chronic Disease, University of Liverpool, Liverpool L7 8TX, UK; 2 Unilever, Colworth Science Park, Sharnbrook, Bedford MK44 1LQ, UK; 3 Department of Nutrition, University of California, Davis, CA 95616, USA

**Keywords:** Jurkat cells, Curcumin, Resveratrol, Isorhamnetin, TNF*α*

## Abstract

Diets rich in fruits and vegetables (FV), which contain (poly)phenols, protect against age-related inflammation and chronic diseases. T-lymphocytes contribute to systemic cytokine production and are modulated by FV intake. Little is known about the relative potency of different (poly)phenols in modulating cytokine release by lymphocytes. We compared thirty-one (poly)phenols and six (poly)phenol mixtures for effects on pro-inflammatory cytokine release by Jurkat T-lymphocytes. Test compounds were incubated with Jurkat cells for 48 h at 1 and 30 µm, with or without phorbol ester treatment at 24 h to induce cytokine release. Three test compounds that reduced cytokine release were further incubated with primary lymphocytes at 0·2 and 1 µm for 24 h, with lipopolysaccharide added at 5 h. Cytokine release was measured, and generation of H_2_O_2_ by test compounds was determined to assess any potential correlations with cytokine release. A number of (poly)phenols significantly altered cytokine release from Jurkat cells (*P*<0·05), but H_2_O_2_ generation did not correlate with cytokine release. Resveratrol, isorhamnetin, curcumin, vanillic acid and specific (poly)phenol mixtures reduced pro-inflammatory cytokine release from T-lymphocytes, and there was evidence for interaction between (poly)phenols to further modulate cytokine release. The release of interferon-*γ* induced protein 10 by primary lymphocytes was significantly reduced following treatment with 1 µm isorhamnetin (*P*<0·05). These results suggest that (poly)phenols derived from onions, turmeric, red grapes, green tea and açai berries may help reduce the release of pro-inflammatory mediators in people at risk of chronic inflammation.

(Poly)phenols, comprising flavonoids and related compounds, are produced by plants that are widely consumed in the human diet. A high dietary intake of fruits and vegetables, which are rich in (poly)phenols, has been linked through epidemiological studies with reduced risk for diseases that are associated with age-related chronic inflammation, including CVD^(^ ^1^ ^–^ ^3^ ^)^, type II diabetes^(^ ^4^ ^,^ ^5^ ^)^, cancers^(^ ^6^ ^–^ ^9^ ^)^, Alzheimer’s disease^(^ ^10^ ^)^ and Parkinson’s disease^(^ ^11^ ^)^. Supplementation with (poly)phenol-rich foods or purified (poly)phenols has been reported to reduce levels of pro-inflammatory cytokines in the human circulation, including TNF*α* and IL-6, cytokines that have been implicated in the pathogenesis of chronic inflammatory diseases^(^ ^12^ ^–^ ^14^ ^)^. T-lymphocytes significantly contribute to the release of pro- and anti-inflammatory cytokines both into the circulation and within tissues. Dietary (poly)phenols may influence the behaviour of T-lymphocytes and other cells via redox signalling, that is the production or quenching of free radicals, or by interactions with specific proteins^(^ ^15^ ^)^. The production of H_2_O_2_ by (poly)phenols through autoxidation catalysed by transition metals is known to influence the behaviour of cells in culture^(^ ^16^ ^,^ ^17^ ^)^, but the effects of (poly)phenol-induced H_2_O_2_ generation on T-lymphocytes and the relevance of this in the modulation of cytokine release are not well understood. A majority of (poly)phenols have low bioavailability *in vivo* as parent compounds^(^ ^18^ ^,^ ^19^ ^)^; therefore, recent interest has been focused on metabolites of dietary (poly)phenols, including the methylated, glucuronidated, and sulphated conjugates that are produced by intestinal and liver cells, and the aromatic acids produced by the colonic bacterial flora^(^ ^20^ ^–^ ^22^ ^)^.

We aimed to compare thirty-one individual (poly)phenols for their effects on the release of inflammatory cytokines by Jurkat CD4^+^ T-lymphocytes. We examined some key dietary (poly)phenols and a number of metabolites, including specifically a substantial number of low molecular weight colonic catabolites. Following this initial screen, three anti-inflammatory compounds were selected for further investigation of their effects on cytokine release by primary peripheral blood mononuclear cell (PBMC)-derived human lymphocytes. We further aimed to assess the generation of H_2_O_2_ in cell culture media by these (poly)phenols and to assess whether H_2_O_2_ generation significantly influences cytokine release or growth of Jurkat CD4^+^ T-lymphocytes *in vitro*. The question of the most appropriate doses of (poly)phenols to be used in *in vitro* studies is controversial within the field. Following dietary intake, (poly)phenols and the individual metabolites/catabolites can be detected in the circulation with *C*_max_ values generally at nm or low µm concentrations, whereas following supplementation with purified compounds or enriched food extracts values of up to 20–50 µm have been reported. For an individual with a typical dietary intake of multiple fruits and vegetables, numerous (poly)phenol metabolites/catabolites coexist in the circulation at any one time. Whether mixtures of different (poly)phenols, each present at low doses, interact in their effects on cytokine release by lymphocytes has not been clear. We therefore also investigated the effects of six different mixtures of (poly)phenols on the release of pro-inflammatory cytokines by Jurkat T-lymphocytes, in order to determine whether multiple (poly)phenols at lower doses have additive or potentially synergistic effects compared with those of single (poly)phenols.

## Methods

### Chemicals and materials

**Fig. 1:**
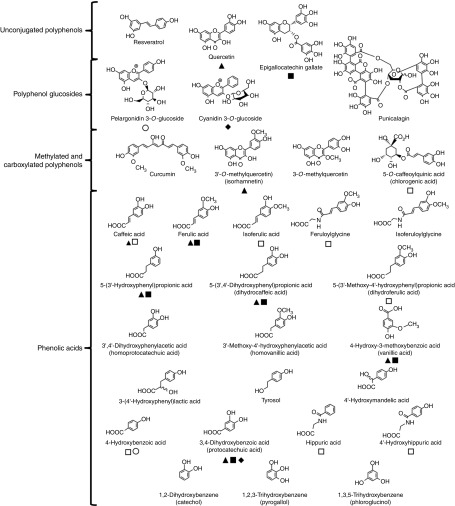
Structures of (poly)phenols used in cell culture experiments. Confirmed metabolic relationships are shown: 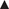, metabolite of quercetin^(^ ^23^ ^,^ ^24^ ^)^; 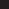, metabolite of (–)-epigallocatechin-3-*O*-gallate^(^ ^25^ ^,^ ^26^ ^)^; 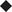, metabolite of cyanidin-3-*O*-glucoside^(^ ^27^ ^)^; 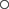, metabolite of pelargonidin-3-*O*-glucoside^(^ ^28^ ^)^; and 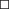, metabolite of chlorogenic acid^(^ ^29^ ^)^. The phenolic catabolite structures are approximately ordered from those produced in the small intestine (top) to those derived in the proximal gastrointestinal tract via colonic catabolism (bottom).

Reagents were purchased from Sigma-Aldrich unless stated otherwise. Sigma-Aldrich also supplied resveratrol, quercetin, isorhamnetin, 3-*O*-methylquercetin, curcumin, (–)-epigallocatechin-3-*O*-gallate, pelargonidin-3-*O*-glucoside, cyanidin-3-*O*-glucoside, chlorogenic acid (5-*O*-caffeoylquinic acid), punicalagin, phloroglucinol (1,3,5-trihydroxybenzene), pyrogallol (1,2,3-trihydroxybenzene), catechol (1,2-dihydroxybenzene), protocatechuic acid (3,4-dihydroxybenzoic acid), 4-hydroxybenzoic acid, homoprotocatechuic acid (3',4'-dihydroxyphenylacetic acid), vanillic acid (3-methoxy-4-hydroxybenzoic acid), homovanillic acid (3'-methoxy-4'-hydroxyphenylacetic acid), 4'-hydroxyphenylacetic acid, 4'-hydroxymandelic acid, 5-(3'-hydroxyphenyl) propionic acid, 3-(4'-hydroxyphenyl) lactic acid, caffeic acid, dihydrocaffeic acid (3-(3',4'-dihydroxyphenyl) propionic acid), ferulic acid, isoferulic acid, dihydroferulic acid (3-(3'-methoxy-4'-hydroxyphenyl) propionic acid), hippuric acid and tyrosol. 4'-Hydroxyhippuric acid was obtained from Bachem Ltd. Feruloylglycine and isoferuloylglycine were generous gifts from Professor Takao Yokota (Teikyo University). Fig. 1 shows the structures of the compounds tested and highlights the metabolic relationships between the compounds.

### Cell culture and treatments

Jurkat E6·1 human CD4^+^ T-lymphocytes obtained from the Health Protection Authority, UK, were cultured routinely at 37°C in a humidified 5 % CO_2_ incubator in Roswell Park Memorial Institute (RPMI)-1640 medium containing phenol red supplemented with 10 % fetal bovine serum (FBS) and 2 mm-l-glutamine. Jurkat cells were seeded at 2×10^6^ cells/ml in a forty-eight-well plate (500 µl culture volume) and treated with each test compound at 1 or 30 μmol/l by dilution from 5 mm stocks in dimethyl sulfoxide (DMSO). The same volume of DMSO only was added to vehicle control wells. After 24 h of incubation with test compounds, 25 ng/ml phorbol 12-myristate 13-acetate (PMA) by 1:1000 dilution from a 25 µg/ml DMSO stock solution and 5 μg/ml phytohaemagglutinin (PHA) by 1:1000 dilution from a 5 mg/ml stock solution in water were added to some wells, and vehicle controls were treated with DMSO at 1:1000 dilution. Typically, the equivalent volume of DMSO added to each well was 0·5 µl. Co-treatment with PMA (a protein kinase C activator) and PHA (a T-cell receptor cross-linking agent) has been widely used to stimulate cytokine production by T-lymphocytes^(^ ^23^ ^)^. Following PMA/PHA treatment, plates were incubated for 24 h at 37°C and 5 % CO_2_. Test compounds were not removed during PMA/PHA treatment, and thus the total incubation period with each (poly)phenol was 48 h. Each treatment was performed in quadruplicate alongside matched vehicle controls. Mixed (poly)phenol treatments were conducted using the same protocol, except that each of the four compounds in each mixture was added at either 0·25 or 7·5 µm, thus achieving either a 1 or 30 µm total concentration of (poly)phenols.

### Measurement of viable cell number

Following incubations with test compounds and stimulation agents, cell numbers were quantified by transferring 100 μl cell suspension from each well to a flat-bottomed, clear, ninety-six-well plate. A sample of 20 ml of 3-(4,5-dimethylthiazol-2-yl)-5-(3-carboxymethoxyphenyl)-2-(4-sulfophenyl)-2H-tetrazolium, inner salt and phenazine methosulfate reagent (Promega) was added to each well and the plate was incubated for 90 min at 37°C and 5 % CO_2_. Following incubation, absorbance was read at 490 nm on a BMG Labtech Spectrostar Nano plate reader. Viable cell numbers were estimated from a standard curve determined by haemocytometer counting with 0·02 % (w/v) trypan blue stain.

### Primary lymphocyte isolation, cell culture and treatment

Primary human lymphocytes were obtained from a healthy 45-year-old female donor after obtaining written consent, by withdrawal of 50 ml blood from the antecubital vein using a butterfly needle and syringe. The procedure was approved by the National Research Ethics service, UK (reference 11/NW/0313), as well as by the University of Liverpool and Royal Liverpool and Broadgreen University Hospitals NHS Trust ethics committees. Whole blood was separated over Ficoll-Paque Premium density gradient medium (GE Healthcare Life Sciences) by centrifugation at 370 ***g*** for 30 min without rotor braking. PBMC were isolated from the buffy layer, centrifuged again at 370 ***g*** for 10 min without rotor braking, then resuspended in FBS containing 10 % DMSO and cryogenically stored. The PBMC were later thawed, washed once in medium (RPMI-1640 supplemented with 10 % FBS and 2 mmol/l of l-glutamine) to remove DMSO, resuspended in the same medium and then cultured at 37°C and 5 % CO_2_ in a T25 tissue culture flask laid horizontally. After 5 d of incubation to allow monocytes to adhere to the culture flask, the lymphocyte-enriched suspension cells were seeded to a forty-eight-well plate at 2×10^6^ cells/ml in 150 µl total volume. After 24 h of incubation at 37°C/5 % CO_2_, the lymphocytes were treated with test compounds (resveratrol, isorhamnetin and curcumin) diluted from 5 mmol/l DMSO stocks to a final concentration of either 0·2 or 1 µmol/l, or the same volume of DMSO only as the vehicle control. Lymphocytes were incubated for 5 h at 37°C/5 % CO_2_; next, 20 ng/ml lipopolysaccharide (LPS) was added without removing media and the cells were incubated for a further 19 h. Following the total 24 h of incubation, cell numbers and viability were determined by trypan blue staining (0·02 % w/v final concentration trypan blue; TC-20 automated cell counter, Bio-Rad) and media were harvested for quantification of cytokines.

### Quantification of cytokines in lymphocyte cell culture media

Cells were pelleted by centrifugation at 400 ***g*** for 5 min. The culture media supernatants were immediately frozen at −80°C and remained frozen until they were thawed for analysis using a BioPlex 200 multi-protein analysis platform (Bio-Rad) and BioPlex bead sets targeting the human cytokine analytes detailed in Table 1. The analysis was performed according to the manufacturer’s instructions. All reagents were purchased from Bio-Rad.

**Table 1:** Cytokines induced by phorbol 12-myristate 13-acetate/phytohaemagglutinin (PMA/PHA) treatment of Jurkat CD4^+^ T-lymphocytes (Mean values with their standard errors)

	Untreated control (pg/ml)		DMSO vehicle control (pg/ml)		24 h PMA/PHA stimulated (pg/ml)	
Cytokines	Mean	sem	Mean	sem	Mean	sem
Eotaxin-1	ND		2·85	1·47	0·48	0·56
GM-CSF	ND		ND		5·81*	0·39
IFN-*γ*	ND		ND		ND	
IL-1*β*	ND		ND		ND	
IL-2	13·45	1·39	14·40	1·41	6969·69*	303·95
IL-4	21·55	0·35	ND		1·86	5·67
IL-6	ND		ND		ND	
IL-7	0·3	0·06	0·80	0·66	3·52	0·66
IL-8	35·25	2·88	34·22	2·32	1612·68*	92·71
IL-12p70	ND		ND		ND	
IL-13	ND		ND		7·68*	1·52
MCP-1	ND		ND		ND	
TNF*α*	ND		ND		259·55*	12·36

GM-CSF, granulocyte macrophage-colony stimulating factor; IFN, interferon; MCP, monocyte chemoattractant protein.

* *P*<0·05 compared with DMSO vehicle control.

Cytokine concentrations were calculated by comparing raw fluorescence emission values with a standard curve diluted from a solution of mixed recombinant cytokine proteins (Bio-Rad). Cytokine concentrations were then normalised for the number of viable cells in the culture at the end of treatment, as quantified by trypan blue exclusion assay. Each time a Jurkat cell treatment experiment was performed, four test compounds were assayed together with a matched vehicle control (either DMSO alone or DMSO with PMA/PHA stimulation at 24 h, as appropriate). Data were normalised to yield percentage changes from vehicle controls by dividing values measured from (poly)phenol-treated cells by the value measured from the matched vehicle control and multiplying by 100. To ascertain whether mixtures of (poly)phenols may synergistically modulate Jurkat T-lymphocyte cytokine release, six mixtures were analysed, each comprised of four (poly)phenols. The results obtained following treatment of Jurkat T-lymphocytes with the (poly)phenol mixtures were compared with the averaged effects of those individual compounds. The total concentration of mixed (poly)phenols and their metabolites was constant at 1 or 30 µmol/l to enable closer comparison with the individual compound treatments.

### Luminescent plate reader analysis of H_2_O_2_ generation

Using an opaque, black, ninety-six-well plate, 5 mm-DMSO stock solutions of thirty-two different (poly)phenols (the same thirty-one compounds tested for anti-inflammatory effects in the Jurkat cell model, plus homovanillic acid) were diluted to a final concentration of 30 µm in 200 µl of working solution (1:1 solution of RPMI-1640 and Amplex Red (10-acetyl-3,7-dihydroxyphenoxazine)-HRP reagent; Invitrogen). A DMSO-only control was used as blank. Fluorescence was measured at 5-min intervals for 24 h with a Fluostar Omega plate reader (BMG Labtech) using an excitation wavelength of 570 nm and an emission wavelength of 585 nm. Kinetic reactions were quantified from the gradient of the linear phase of each reaction and converted into the rates of H_2_O_2_ generation (nmol/min) by comparison with H_2_O_2_ standards.

### Statistical analysis

Statistical analyses were performed using SPSS version 20 (IBM) using raw data for cell number and H_2_O_2_ generation and data normalised to cell number for cytokine measurements. Jurkat CD4^+^ T-lymphocytes and primary PBMC-derived lymphocytes treated with (poly)phenols were compared with matched vehicle controls by one-way ANOVA with Dunnett’s *post hoc* test. The predicted effects of mixtures of test compounds on cytokine release by Jurkat CD4^+^ T-lymphocytes were compared with the observed effects by one-way ANOVA with Tukey’s *post hoc* test. All the statistical tests were performed at *α*=0·05.

## Results

### Phorbol 12-myristate 13-acetate/phytohaemagglutinin 24-h treatment stimulates the release of pro-inflammatory cytokines by Jurkat CD4^+^ T-lymphocytes

Jurkat CD4+ T-lymphocytes were treated with the protein kinase C activator PMA and the plant-derived lymphocyte mitogen PHA for 24 h to induce an inflammatory response and cytokine release. The PMA/PHA-stimulated cells showed a significant increase in the release of several pro-inflammatory cytokines compared with vehicle control treatments (Table 1). We chose to focus on IL-2, IL-8 and TNF*α* for further experiments because of the relative magnitude of their induction by PMA/PHA treatment and the well-defined roles of each of these proteins as physiologically important pro-inflammatory cytokines.

### Polyphenol catabolites modulate growth and cytokine release by Jurkat CD4^+^ T-lymphocytes

**Fig. 2:**
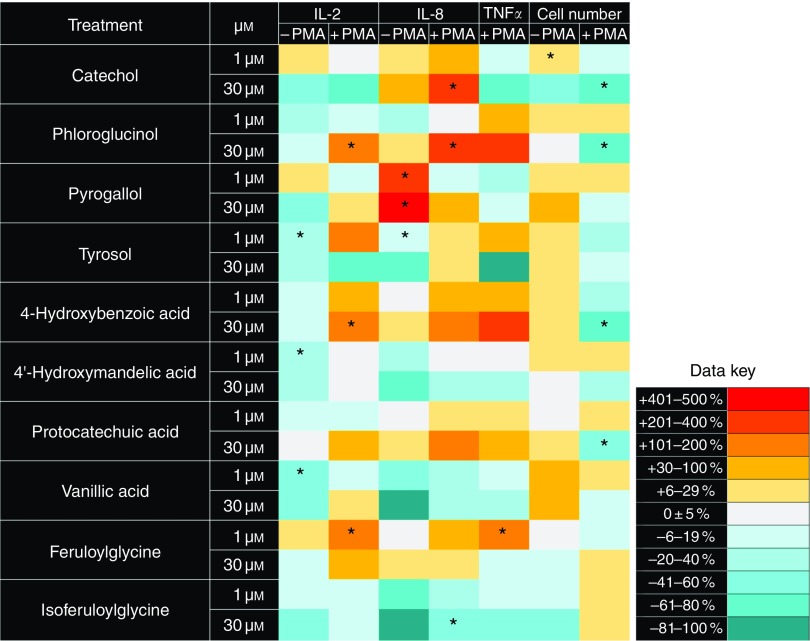
Heat map showing the effects of phenolic acids on cytokine release and growth by Jurkat CD4^+^ T-lymphocytes. Compounds are ordered by molecular weight from the lowest weight (top) to the highest weight (bottom). Data are presented as percentage differences from matched vehicle controls following 48 h of treatment. Treatment and control experiments were performed with or without 25 ng/ml phorbol 12-myristate 13-acetate (PMA) and 5 µg/ml phytohaemagglutinin (PHA) stimulation at 24 h. TNF*α* could not be measured in the absence of PMA/PHA stimulation. * Mean value was significantly different compared with vehicle controls (*P*<0·05; one-way ANOVA with Dunnett’s *post hoc* test). No significant effects were observed following treatment with caffeic acid, ferulic acid, isoferulic acid, 5-(3'-hydroxyphenyl) propionic acid, 5-(3',4'-dihydroxyphenyl) propionic acid, 5-(3'-methoxy-4'-hydroxyphenyl) propionic acid, homoprotocatechuic acid, 3-(4' hydroxyphenyl) lactic acid, hippuric acid or 4'-hydroxyhippuric acid. Examples of the data from which these heat maps are derived are provided in the online Supplementary data to allow an assessment of the variability observed in these studies.

After treatment with colonic catabolites of polyphenols (phenolic acids) for 48 h, we measured the density of viable Jurkat CD4^+^ T-lymphocytes in culture and the concentrations of the pro-inflammatory cytokines IL-2, IL-8 and TNF*α* in the cell culture media. Comparisons between (poly)phenol treatments and vehicle controls revealed multiple effects on growth and cytokine release (Fig. 2). The low molecular weight (110–139 g/mol) catabolites catechol, phloroglucinol and 4-hydroxybenzoic acid caused significant declines in cell number (*P*<0·05; one-way ANOVA with Dunnett’s *post hoc* test) and significantly induced release of IL-2, IL-8 and TNF*α* (*P*<0·05; one-way ANOVA with Dunnett’s *post hoc* test), whereas tyrosol significantly decreased IL-2 and IL-8 release from non-stimulated cells at 1 µmol/l (*P*<0·05; one-way ANOVA with Dunnett’s *post hoc* test). Mid-molecular weight catabolites (168–195 g/mol) reduced pro-inflammatory cytokine release, with significant reduction in IL-2 release from non-stimulated cells by 1 µmol/l 4'-hydroxymandelic acid and vanillic acid (*P*<0·05; one-way ANOVA with Dunnett’s *post hoc* test). Higher molecular weight test compounds (196–252 g/mol) showed mixed effects: feruloylglycine at 1 µmol/l significantly increased the release of IL-2 and TNF*α*, and isoferuloylglycine at 30 µmol/l significantly reduced IL-8 release from PMA/PHA-stimulated Jurkat CD4^+^ T-lymphocytes (*P*<0·05; one-way ANOVA with Dunnett’s *post hoc* test).

### Polyphenols modulate growth and pro-inflammatory cytokine release by Jurkat CD4^+^ T-lymphocytes

**Fig. 3:**
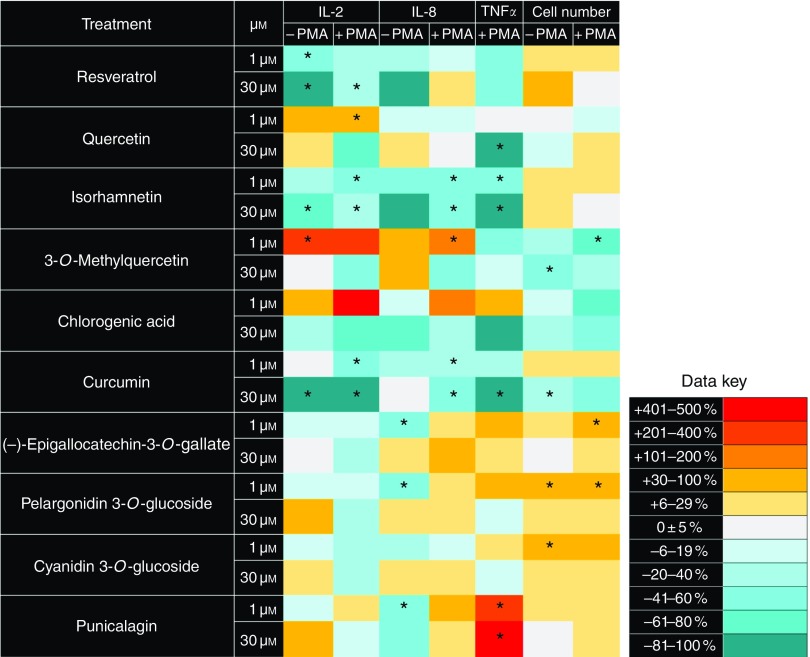
Heat map showing the effects of polyphenols on cytokine release and growth by Jurkat CD4^+^ T-lymphocytes. Compounds are ordered by molecular weight from the lowest weight (top) to the highest weight (bottom). Data are presented as percentage differences from matched vehicle controls following 48 h of treatment. Treatment and control experiments were performed with or without 25 ng/ml phorbol 12-myristate 13-acetate (PMA) and 5 µg/ml phytohaemagglutinin (PHA) stimulation at 24 h. TNF*α* could not be measured in the absence of PMA/PHA stimulation. * Mean value was significantly different compared with vehicle controls (*P*<0·05; one-way ANOVA with Dunnett’s *post hoc* test). Examples of the data from which these heat maps are derived are provided in the online Supplementary data to allow an assessment of the variability observed in these studies.

Treatment with different polyphenols had varied effects on growth and pro-inflammatory cytokine release by Jurkat CD4^+^ T-lymphocytes (Fig. 3). Three polyphenols – resveratrol, isorhamnetin and curcumin – significantly reduced pro-inflammatory cytokine release at both 1 and 30 µmol/l and in both unstimulated and PMA/PHA-stimulated cells (*P*<0·05; one-way ANOVA with Dunnett’s *post hoc* test). Curcumin treatment at 30 µmol/l led to the greatest reductions in pro-inflammatory cytokine release, with IL-2 release decreased by 96 % and TNF*α* release ablated to undetectable concentrations in PMA/PHA-stimulated cells (*P*<0·05; one-way ANOVA with Dunnett’s *post hoc* test). The flavan-3-ol (–)-epigallocatechin-3-*O*-gallate, and the anthocyanins pelargonidin-3-*O*-glucoside and cyanidin 3-*O*-glucoside, significantly promoted the growth of Jurkat CD4^+^ T-lymphocytes at 1 µmol/l (*P*<0·05; one-way ANOVA with Dunnett’s *post hoc* test). Chlorogenic acid and 3-*O*-methylquercetin showed some anti-proliferative effects on Jurkat CD4^+^ T-lymphocytes under all treatment conditions, although statistical significance was only achieved for the effects of 3-*O*-methylquercetin (*P*<0·05; one-way ANOVA with Dunnett’s *post hoc* test).

### Mixtures of (poly)phenols interact to modulate cytokine release

**Fig. 4:**
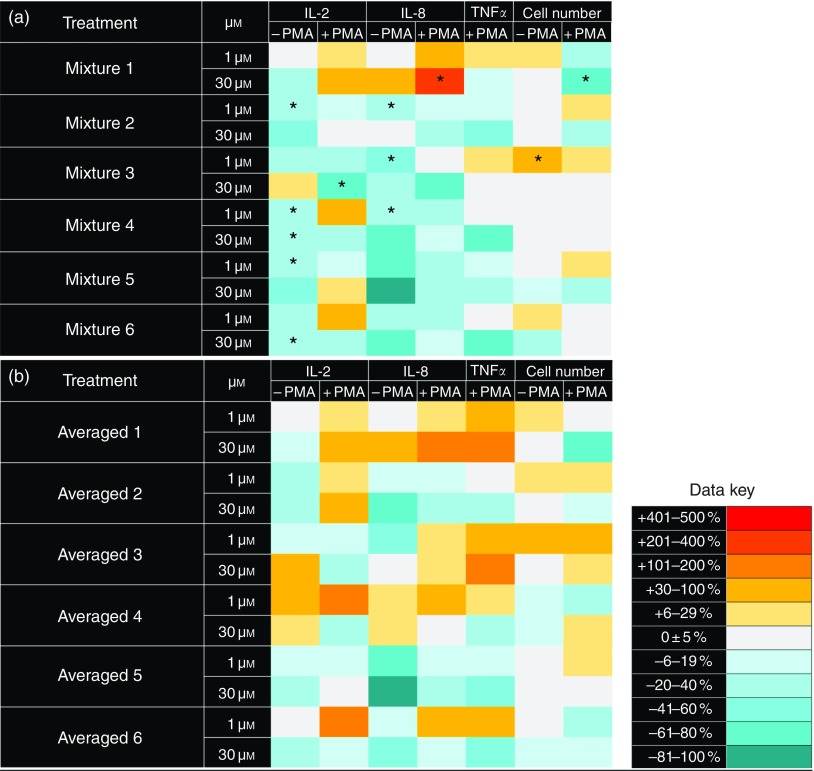
Heat maps showing cytokine release by Jurkat CD4^+^ T-lymphocytes following treatment with mixtures of four (poly)phenols at 1 or 30 µmol/l compared with vehicle controls (a), and mathematical averages of the effects on cytokine release following treatment with the individual compounds (b). Cells were incubated with (poly)phenol mixtures for 48 h, with or without the addition of 25 ng/ml phorbol myristoyl acetate and 5 µg/ml phytohaemagglutinin at the 24-h time point. The mixtures comprised the following: (1) catechol, phloroglucinol, 4-hydroxybenzoic acid and protocatechuic acid; (2) 4'-hydroxymandelic acid, 4-hydroxyphenylacetic acid, 5-(3'-hydroxyphenyl) propionic acid and 3-(4'-hydroxyphenyl) lactic acid; (3) (–)-epigallocatechin-3-*O*-gallate, pelargonidin-3-*O*-glucoside, cyanidin-3-*O*-glucoside and punicalagin; (4) dihydroferulic acid, feruloylglycine, quercetin and 3-*O*-methylquercetin; (5) caffeic acid, ferulic acid, isoferulic acid and isoferuloylglycine; and (6) hippuric acid, tyrosol, 4'-hydroxyhippuric acid and chlorogenic acid. * Mean value was significantly different between the ‘expected’ response predicted by mathematically averaging the effects of treatment with individual compounds and the ‘observed’ response measured following treatment with the mixtures (*P*<0·05; one-way ANOVA with Tukey’s *post hoc* test).

To assess potential interactions between test compounds in modulating cytokine release, we prepared several mixtures of (poly)phenols: mixture 1 representing low-molecular weight colonic catabolites such as catechol, phloroglucinol, 4-hydroxybenzoic acid and protocatechuic acid; mixture 2 representing mid-molecular weight colonic catabolites such as 4'-hydroxymandelic acid, 4'-hydroxyphenylacetic acid, 5-(3'-hydroxyphenyl) propionic acid and 3-(4'-hydroxyphenyl) lactic acid; mixture 3 representing a mixture of dietary polyphenols such as (–)-epigallocatechin-3-*O*-gallate, pelargonidin-3-*O*-glucoside, cyanidin-3-*O*-glucoside and punicalagin; mixture 4 representing polyphenols and high molecular weight hydroxycinnamate metabolites, such as dihydroferulic acid, feruloylglycine, quercetin and 3-*O*-methylquercetin; mixture 5 representing a mixture of hydroxycinnamate derivatives derived from chlorogenic acid after the consumption of coffee, such as caffeic acid, ferulic acid, isoferulic acid and isoferuloylglycine; and mixture 6 representing compounds derived from apple cider, such as hippuric acid, tyrosol, 4'-hydroxyhippuric acid and chlorogenic acid. We measured cytokine release from Jurkat CD4^+^ T-lymphocytes following 48 h of incubation with each mixture at a total concentration of 1 or 30 µmol/l, with or without stimulation with PMA/PHA at 24 h. For comparison, the results previously obtained for cytokine release following treatment with each individual compound were averaged to generate a prediction of the effects expected if there were no interactions between compounds (i.e. a null hypothesis that there were no synergistic effects). The results illustrated in Fig. 4 indicate that five of the six test mixtures significantly reduced pro-inflammatory cytokine release in comparison with results anticipated from simple addition of the effects observed for individual compounds (*P*<0·05; one-way ANOVA with Dunnett’s *post hoc* test).

### Anti-inflammatory polyphenols modulate cytokine release by peripheral blood mononuclear cell-derived human lymphocytes

**Fig. 5:**
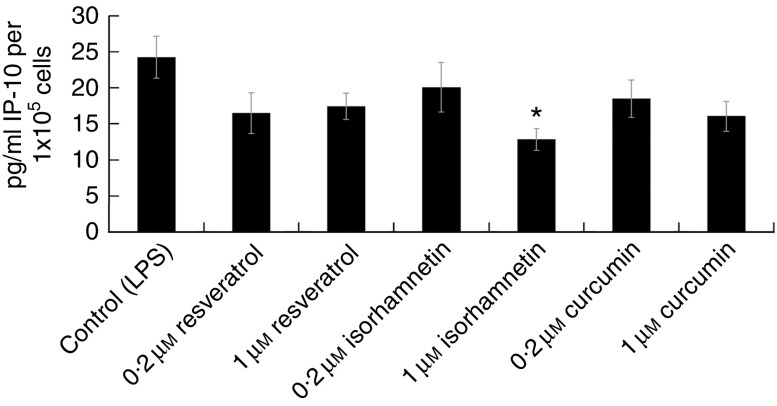
Cytokine release by lymphocytes from a healthy donor following treatment for 24 h with the polyphenols resveratrol, isorhamnetin and curcumin with the addition of 20 ng/ml lipopolysaccharide (LPS) at the 5-h time point. Isorhamnetin at 1 µmol/l significantly reduced interferon-*γ* induced protein 10 (IP-10) release compared with the matched DMSO vehicle control (*P*<0·05; one-way ANOVA with Dunnett’s *post ho*c test). *Groups that significantly differed from the control group (one-way ANOVA with Dunnett’s *post hoc* test; *P*<0·05). There were no significant differences between groups for other cytokines. Data for the other cytokines are available in the Online Supplementary Fig. S2. LVC, LPS and vehicle control; 0·2 and 1 denote concentrations in µmol/l.

We investigated the polyphenols resveratrol, isorhamnetin and curcumin, which had been identified to be the most effective in reducing cytokine release from among the panel of (poly)phenols that we screened in Jurkat CD4^+^ T-lymphocytes, to determine whether their inhibitory effects on pro-inflammatory cytokine release were sustained in PBMC-derived human lymphocytes (Fig. 5). The results suggest trends (i.e. *P*<0·09) towards decreased IL-6, interferon-*γ* induced protein 10 (IP-10) and TNF*α* release following treatment with resveratrol, isorhamnetin and curcumin at 0·2 or 1 µm. The reduction in IP-10 release after treatment with 1 µm isorhamnetin was statistically significant (*P*<0·05, one-way ANOVA with Dunnett’s *post hoc* test).

### (Poly)phenols produce H_2_O_2_ in cell culture media

**Fig. 6:**
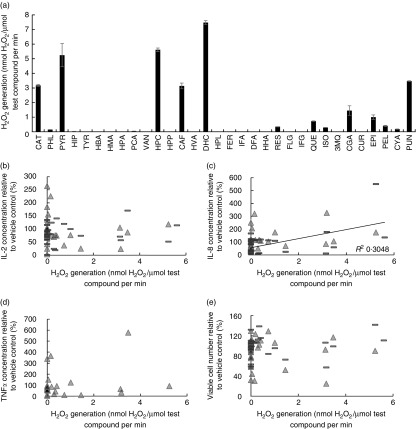
Generation of H_2_O_2_ by (poly)phenols at 30 µmol/l concentration in Roswell Park Memorial Institute (RPMI)-1640 medium containing 10 % fetal calf serum and phenol red (a), and relationships with Jurkat CD4^+^ T-lymphocyte pro-inflammatory cytokine release and cell growth (b–e). H_2_O_2_ production was measured by a kinetic reaction between each (poly)phenol incubated at 30 µmol/l with Amplex red reagent, which fluoresces following reaction with H_2_O_2_ (a). Scatter plots were constructed for H_2_O_2_ production against Jurkat CD4^+^ T-lymphocyte cytokine release (b=IL-2, c=IL-8, d=TNF*α*) or cell number (e) after 48 h of treatment, with or without phorbol 12-myristate 13-acetate (PMA)/phytohaemagglutinin (PHA) stimulation to induce cytokine release at the 24 h time point. A linear correlation was identified between H_2_O_2_ production and IL-8 release in non-stimulated cells only (*R* ^2^ 0·3048; trend line shown in figure). 3MQ, 3-*O*-methylquercetin; CAF, caffeic acid; CAT, catechol; CGA, chlorogenic acid; CUR, curcumin; CYA, cyanidin-3-*O*-glucoside; DHC, dihydrocaffeic acid; DFA, dihydroferulic acid; EPI, (–)-epigallocatechin-3-*O*-gallate; FER, ferulic acid; FLG, feruloylglycine; HBA, 4-hydroxybenzoic acid; HHA, 4'-hydroxyhippuric acid; HIP, hippuric acid; HMA, 4'-hydroxymandelic acid; HPA, 4'-hydroxyphenylacetic acid; HPC, homoprotocatechuic acid; HPL, 3-(4'-hydroxyphenyl)lactic acid; HPP, 5-(3'-hydroxyphenyl)propionic acid; HVA, homovanillic acid; IFA, isoferulic acid; IFG, isoferuloylglycine; ISO, isorhamnetin; PCA, protocatechuic acid; PEL, pelargonidin-3-*O*-glucoside; PHL, phloroglucinol; PUN, punicalagin; PYR, pyrogallol; QUE, quercetin; RES, resveratrol; TYR, tyrosol; VAN, vanillic acid. Compounds are ordered from least (CAT) to highest (PUN) molecular weight along the *x*-axis. ■, Data from non-stimulated cells; ▲, data from PMA/PHA-stimulated cells.

Some (poly)phenols have been reported to generate H_2_O_2_ in cell culture media^(^ ^16^ ^,^ ^17^ ^)^. To quantify H_2_O_2_ production, we conducted kinetic spectrophotometry assays using Amplex red reagent, which is converted to fluorescent resorufin following oxidation by H_2_O_2_. Production of H_2_O_2_ was detected for sixteen of the thirty-two test compounds (Fig. 6(a)). H_2_O_2_ production was detected from the hydroxybenzene derivatives catechol and pyrogallol, the phenylacetic acid homoprotocatechuic acid, the hydroxycinnamates caffeic acid and dihydrocaffeic acid, and the ellagitannin punicalagin, whereas other test compounds produced no detectable levels of H_2_O_2_. Comparisons were also made to assess the effects of phenol red in the culture media, which showed that rates of H_2_O_2_ production were 24 (sem 2) % lower in RPMI-1640 medium containing phenol red than in RPMI-1640 medium without phenol red (data not shown in detail).

### Production of H_2_O_2_ by (poly)phenols does not strongly correlate with cytokine release by CD4^+^ T-lymphocytes

Correlation assays were performed between the H_2_O_2_ production by test compounds and their effects on Jurkat CD4^+^ T-lymphocyte growth and pro-inflammatory cytokine release (Fig. 6(b)–(e)). These data indicate a potential correlation between H_2_O_2_ production and IL-8 release in non-PMA/PHA-stimulated cells (*R* ^2^ 0·3048).

## Discussion

In these studies, we evaluated a panel of thirty-one (poly)phenols for potential anti-inflammatory activity using a human T-lymphocyte model of cytokine production. The compounds tested were grouped by their molecular weight (Fig. 2 & 3), as the lower molecular weight compounds tended to be the colonic catabolites (phenolic acids). We observed substantial inhibition of cytokine release primarily by the parent polyphenols, several of which modified cytokine release and proliferation by T-lymphocytes under both baseline and activated (PMA/PHA stimulated) conditions. We also investigated mixtures of (poly)phenols, and our data show that five of the six mixtures of test compounds had greater anti-inflammatory effects than that predicted under our null hypothesis based on the effects of the individual compounds. We further explored the effects of three of the dietary polyphenols showing anti-inflammatory effects in Jurkat cells in healthy human lymphocytes and found that isorhamnetin reduced pro-inflammatory cytokine release from LPS-stimulated lymphocytes at 1 µm. (Poly)phenols have been proposed to reduce the risk of developing chronic diseases during ageing by modulating inflammatory responses and the production of pro-inflammatory cytokines in multiple tissues^(^ ^25^ ^)^.

A number of previous studies have examined the potential effects of various polyphenols on cytokine release^(^ ^28^ ^–^ ^31^ ^)^, and a number of dietary components including curcumin, resveratrol, genistein and epigallocatechin have been shown to modulate the release of pro-inflammatory cytokines from cells in culture^(^ ^30^ ^)^. Various mechanisms of action have been proposed including inhibition of NF-*κ*B, inhibition of prostanoids, inhibition of AMP-activated protein kinase (AMPK) and mitogen-activated protein kinase (MAPK) pathways and antioxidant effects^(^ ^31^ ^)^. There has been a great deal of interest in the bioavailability of dietary polyphenols, as the non-metabolised compounds are only found in the circulation at nm to low µm concentrations. Compounds such as flavonoids are absorbed in the small intestine and appear in the circulation as glucuronoids, sulphates and methylated metabolites, but these are rapidly removed from the bloodstream^(^ ^32^ ^)^; however, substantial amounts of the unconjugated compounds pass into the colon where they are converted to lower molecular weight catabolites such as phenolic acids by colonic microflora^(^ ^33^ ^)^. Relatively little is known about the potential anti-inflammatory effects of these lower molecular weight phenolic acids following absorption, and this was a major aim of the present study.

Polyphenols were routinely examined at final concentrations of either 1 or 30 µm, reflecting concentrations that may potentially be achieved in the circulation through ingestion of foodstuffs or supplements, respectively. Our data showed that several (poly)phenol treatments modulated the release of pro-inflammatory cytokines (IL-2, IL-8 and TNF*α*) from Jurkat human CD4^+^ T-lymphocytes. The Jurkat cell line was originally derived from an adolescent male with acute lymphoblastic leukaemia and they are widely used as a model for pro-inflammatory cytokine release^(^ ^34^ ^)^. Jurkat CD4^+^ T-lymphocytes were incubated with test compounds at 1 and 30 µm either non-stimulated or stimulated with PMA/PHA. We were interested to investigate both lower and higher dose ranges based on previous reports of differential effects between low- and high-dose (poly)phenols *in vivo* ^(^ ^35^ ^)^. The most potent anti-inflammatory compounds from our experiments in Jurkat CD4^+^ T-lymphocytes were isorhamnetin (a flavonol that occurs as a glycoside in apples, onions and green tea), curcumin (from the Indian spice turmeric) and resveratrol (which is present in the skin of red, purple and black grapes and in especially high concentrations in Itadori tea).

The low molecular weight phenolic acids (Fig. 2) were generally less effective at reducing cytokine release than the unconjugated polyphenols, although some reductions in Il-2 release were seen with vanillic acid, tyrosol and 4-hydroxymandelic acid. Vanillic acid is a catabolite of multiple dietary polyphenols, including those found in wheat and blackcurrant juice^(^ ^36^ ^)^, and is also present at high concentrations in açai berries^(^ ^37^ ^,^ ^38^ ^)^. Vanillic acid significantly reduced IL-2 release from non-stimulated Jurkat CD4^+^ T-lymphocytes at 1 µmol/l, which is within the range of concentrations previously reported in human plasma^(^ ^36^ ^)^, suggesting that vanillic acid may be a physiologically relevant anti-inflammatory metabolite of dietary polyphenols.

Following ingestion of (poly)phenol-rich foodstuffs, a range of (poly)phenols, conjugates and catabolites are absorbed into the circulation. It is not understood whether the exposure of CD4^+^ T-lymphocytes to mixtures of these compounds may elicit different responses in comparison with the same compounds applied individually. To begin to address this issue, we treated Jurkat CD4^+^ T-lymphocytes with six different mixtures of (poly)phenols. The mixtures of compounds used were selected as representative of low molecular weight colonic metabolites (mix 1), mid-molecular weight colonic catabolites (mix 2), dietary polyphenols (mix 3), polyphenols and high-molecular weight hydroxycinnamate metabolites (mix 4), hydroxycinnamate derivatives derived from chlorogenic acid produced after the consumption of coffee (mix 5) and compounds potentially derived from apples (mix 6). Of these, five mixtures reduced cytokine release more than that predicted based on the cumulative effects of individual compounds. In contrast, mixture 1 was found to be relatively pro-inflammatory and comprised low molecular weight catabolites that individually induced pro-inflammatory cytokine release. Thus, we speculate that there may be synergistic anti-inflammatory effects of some polyphenols when they are present in foodstuffs, and the data obtained generally support this. Demonstration of true synergy between bioactive materials such as drugs requires a rigorous statistical approach^(^ ^39^ ^)^, which we do not have the dose–response data to undertake for the large group of polyphenols that were studied; however, the data presented appear to indicate the possibility of such synergistic effects and warrant further investigation.

It has been reported that (poly)phenols produce H_2_O_2_ in cell culture media due to autoxidation catalysed by transition metals such as Fe and Cu and that this may influence the responses of cells in culture^(^ ^17^ ^,^ ^40^ ^)^. We measured the production of H_2_O_2_ by different (poly)phenols and related compounds in RPMI-1640 medium containing 10 % FBS using the Amplex red-HRP technique. This assay is specific for H_2_O_2_ in simple solutions and has been widely used to examine H_2_O_2_ release by cells and sub-cellular fractions of cells^(^ ^41^ ^)^. H_2_O_2_ generation was found to vary between compounds, with certain phenolic catabolites producing relatively large amounts of H_2_O_2_ and others producing no detectable H_2_O_2_. Analysis of H_2_O_2_ generation by (poly)phenols in comparison with their chemical structures indicated that, in accordance with theoretical predictions^(^ ^40^ ^)^, molecules with orthohydroxy groups on adjacent carbons of a benzene ring tended to generate H_2_O_2_. However, protocatechuic acid and 3-*O*-methylquercetin, which have benzene ring orthohydroxy groups, generated negligible H_2_O_2_, and moderate H_2_O_2_ generation was detected from isorhamnetin and resveratrol, which do not have orthohydroxy groups, implying that other aspects of the chemical structure also influence H_2_O_2_ generation. The average rate of H_2_O_2_ generation for these compounds in RPMI-1640 medium was 5·6 nmol H_2_O_2_/µmol (poly)phenol per min. We investigated whether H_2_O_2_ generation by each test compound correlated with their effects on the release of IL-2, IL-8 or TNF*α* by Jurkat CD4^+^ T-lymphocytes or the growth of Jurkat cultures. A correlation was identified between H_2_O_2_ production and IL-8 release by CD4^+^ T-lymphocytes that were not stimulated with PMA/PHA. Interestingly, previous studies have indicated that IL-8 production is subject to modulation by redox signalling^(^ ^34^ ^)^ and can be induced by extracellular H_2_O_2_ in epithelial cell lines via activation of the redox-sensitive transcription factors activator protein-1 (AP-1) and NF-*к*B^(^ ^42^ ^,^ ^43^ ^)^. Our results suggest that with PMA/PHA stimulation the influence of H_2_O_2_ on IL-8 release appears to become negligible. IL-2 and TNF*α* release and cell proliferation showed no clear relationship with H_2_O_2_ generation in non-stimulated or PMA/PHA-stimulated Jurkat CD4^+^ T-lymphocytes. Halliwell *et al*.^(^ ^44^ ^,^ ^45^ ^)^ have also pointed out that the absence of any detection of H_2_O_2_ generation by a compound in cell culture cannot be equated to the stability of the compound as some polyphenols were observed to rapidly degrade and autoxidise in the absence of detectable H_2_O_2_ generation. Thus, our studies indicate a lack of correlation between *in vitro* H_2_O_2_ generation and effects of the compound on cytokine release, but do not exclude other cell culture artifacts that may have influenced the data obtained.

We chose to undertake a limited proof-of-principle study to investigate the potential anti-inflammatory effects of isorhamnetin, curcumin and resveratrol in primary PBMC-derived human lymphocytes. Cells were pre-treated with 1 or 30 µm polyphenol for 5 h, followed by stimulation of cytokine release using LPS stimulation and incubation for a total period of 24 h. We observed a significant reduction in IP-10 release following treatment with 1 µm isorhamnetin, and there was also a trend (*P*=0·07) towards reduced TNF*α* release with the same treatment. These studies were undertaken using lymphocytes from a single donor and clearly require confirmation using cells from a larger group of donors, but this approach was considered out with the remit of the present study due to the large variability in cytokine release from lymphocytes obtained from different donors.

In conclusion, we have shown that (poly)phenols modulate the release of cytokines by Jurkat CD4^+^ T-lymphocytes under resting and chemically activated conditions. Some compounds were found to have anti-inflammatory effects, while others were pro-inflammatory, and the effects varied with dose, suggesting that some dietary (poly)phenols may be beneficial for the prevention or management of chronic inflammatory conditions. We identified isorhamnetin, curcumin, resveratrol and vanillic acid as negative regulators of pro-inflammatory cytokine release in Jurkat CD4^+^ T-lymphocytes, and also showed that 1 µm isorhamnetin reduced pro-inflammatory cytokine release by primary human lymphocytes. Use of mixtures of (poly)phenols also suggested that they may act synergistically to modulate cytokine release by Jurkat CD4^+^ T-lymphocytes. Taking into account the main dietary sources of the compounds, our data support a potential anti-inflammatory role for (poly)phenols derived from red, purple and black grapes, turmeric, whole wheat, blackcurrants, apples and onions, and suggest that individuals at risk of chronic inflammation, such as older people, may benefit from supplementing their diets with isorhamnetin, resveratrol, curcumin and vanillic acid or with food sources that yield these bioactive molecules.

## Supplementary material

For supplementary material accompanying this paper visit http://dx.doi.org/10.1017/S0007114516000805. click here to view supplementary material
