# Supplementary material for: Identification of (poly)phenol treatments that modulate the release of pro-inflammatory cytokines by human lymphocytes
Source: Br J Nutr. 2016 Mar 17;115(10):1699–710. doi: 10.1017/S0007114516000805 (PMC4836295; doi:10.1017/S0007114516000805)
Supplement: Supplementary file 1 [file S0007114516000805sup001.zip › S0007114516000805sup001/S0007114516000805sup002.pptx]

## Slide 1
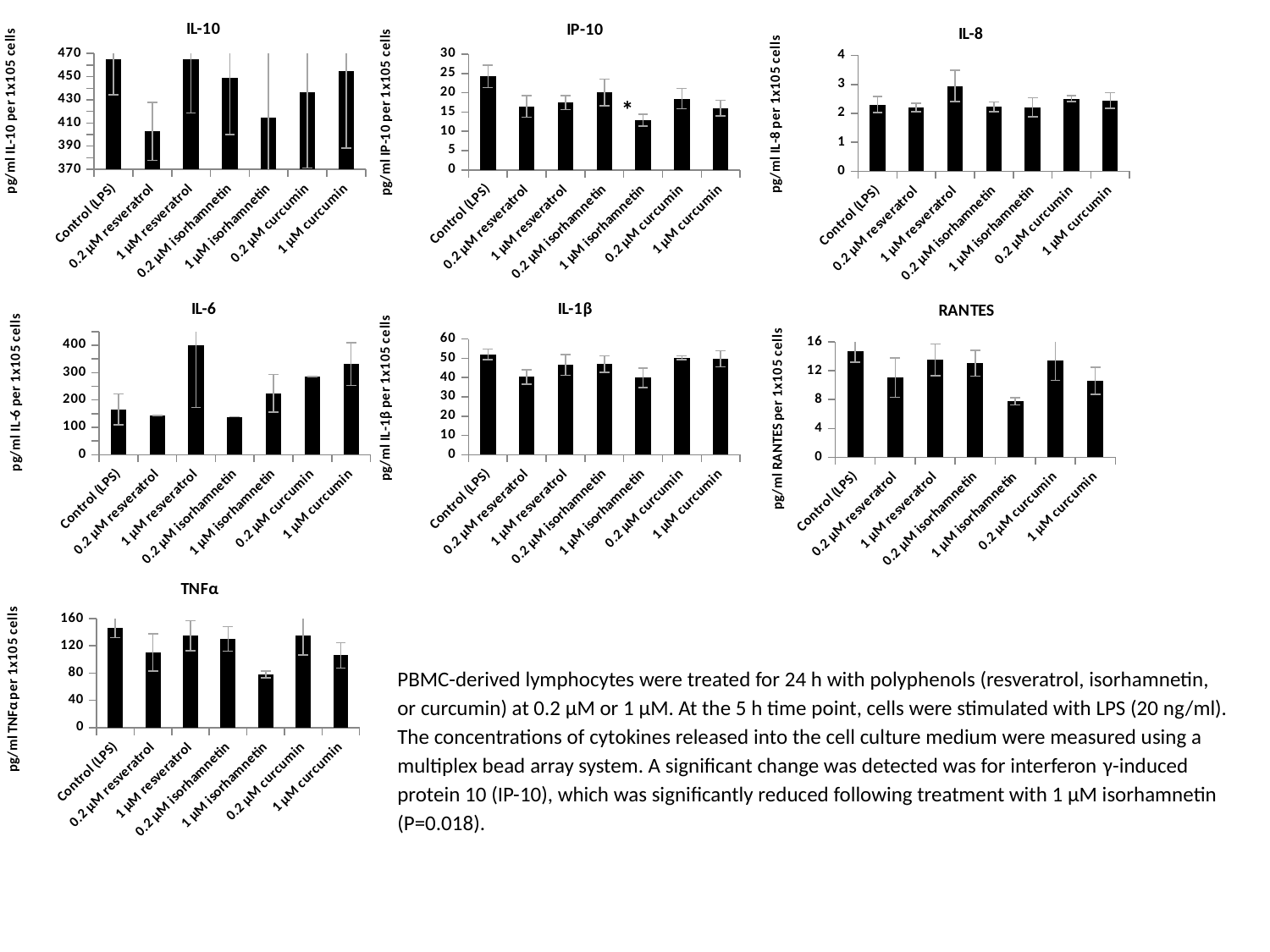

### Chart: IL-10
| Category | Hu IL-10 (56) |
|---|---|
| Control (LPS) | 465.01073265822214 |
| 0.2 µM resveratrol | 402.6930685776595 |
| 1 µM resveratrol | 464.8533613917756 |
| 0.2 µM isorhamnetin | 449.0001457014567 |
| 1 µM isorhamnetin | 414.2732571089984 |
| 0.2 µM curcumin | 436.52516797769795 |
| 1 µM curcumin | 454.7818542956884 |
### Chart: IP-10
| Category | Hu IP-10 (48) |
|---|---|
| Control (LPS) | 24.257770034054523 |
| 0.2 µM resveratrol | 16.49544187531292 |
| 1 µM resveratrol | 17.431597458911796 |
| 0.2 µM isorhamnetin | 20.077929930673427 |
| 1 µM isorhamnetin | 12.87774253317618 |
| 0.2 µM curcumin | 18.478165233772607 |
| 1 µM curcumin | 16.05428912985921 |
### Chart: IL-8
| Category | Hu IL-8 (54) |
|---|---|
| Control (LPS) | 2.3013587775628976 |
| 0.2 µM resveratrol | 2.208066887363993 |
| 1 µM resveratrol | 2.946028127133334 |
| 0.2 µM isorhamnetin | 2.2190144306358137 |
| 1 µM isorhamnetin | 2.207779720402092 |
| 0.2 µM curcumin | 2.5102090152006262 |
| 1 µM curcumin | 2.447538952172752 |*
### Chart: IL-6
| Category | Hu IL-6 (19) |
|---|---|
| Control (LPS) | 166.06686691853596 |
| 0.2 µM resveratrol | 143.72790322580644 |
| 1 µM resveratrol | 400.69581421055295 |
| 0.2 µM isorhamnetin | 138.2350762527233 |
| 1 µM isorhamnetin | 224.57199781149734 |
| 0.2 µM curcumin | 285.78931297709926 |
| 1 µM curcumin | 331.5777379186353 |
### Chart: IL-1β
| Category | Hu IL-1b (39) |
|---|---|
| Control (LPS) | 51.949953338989005 |
| 0.2 µM resveratrol | 40.27790459275387 |
| 1 µM resveratrol | 46.435259391169176 |
| 0.2 µM isorhamnetin | 47.04765928069573 |
| 1 µM isorhamnetin | 39.85918666360745 |
| 0.2 µM curcumin | 50.25322382313603 |
| 1 µM curcumin | 49.62145757654407 |
### Chart: RANTES
| Category | Hu RANTES (37) |
|---|---|
| Control (LPS) | 14.671444149328916 |
| 0.2 µM resveratrol | 11.055045462093567 |
| 1 µM resveratrol | 13.49957075991578 |
| 0.2 µM isorhamnetin | 13.037635153973318 |
| 1 µM isorhamnetin | 7.781035765564411 |
| 0.2 µM curcumin | 13.47665978821197 |
| 1 µM curcumin | 10.623929014139561 |
### Chart: TNFα
| Category | Hu TNF-a (36) |
|---|---|
| Control (LPS) | 146.71444149328917 |
| 0.2 µM resveratrol | 110.55045462093567 |
| 1 µM resveratrol | 134.9957075991578 |
| 0.2 µM isorhamnetin | 130.3763515397332 |
| 1 µM isorhamnetin | 77.81035765564411 |
| 0.2 µM curcumin | 134.7665978821197 |
| 1 µM curcumin | 106.23929014139563 |PBMC-derived lymphocytes were treated for 24 h with polyphenols (resveratrol, isorhamnetin, or curcumin) at 0.2 µM or 1 µM. At the 5 h time point, cells were stimulated with LPS (20 ng/ml). The concentrations of cytokines released into the cell culture medium were measured using a multiplex bead array system. A significant change was detected was for interferon γ-induced protein 10 (IP-10), which was significantly reduced following treatment with 1 µM isorhamnetin (P=0.018).
