# Supplementary figures and images for: Identification of (poly)phenol treatments that modulate the release of pro-inflammatory cytokines by human lymphocytes
Source: Br J Nutr. 2016 Mar 17;115(10):1699–710. doi: 10.1017/S0007114516000805 (PMC4836295; doi:10.1017/S0007114516000805)

## Slide 1
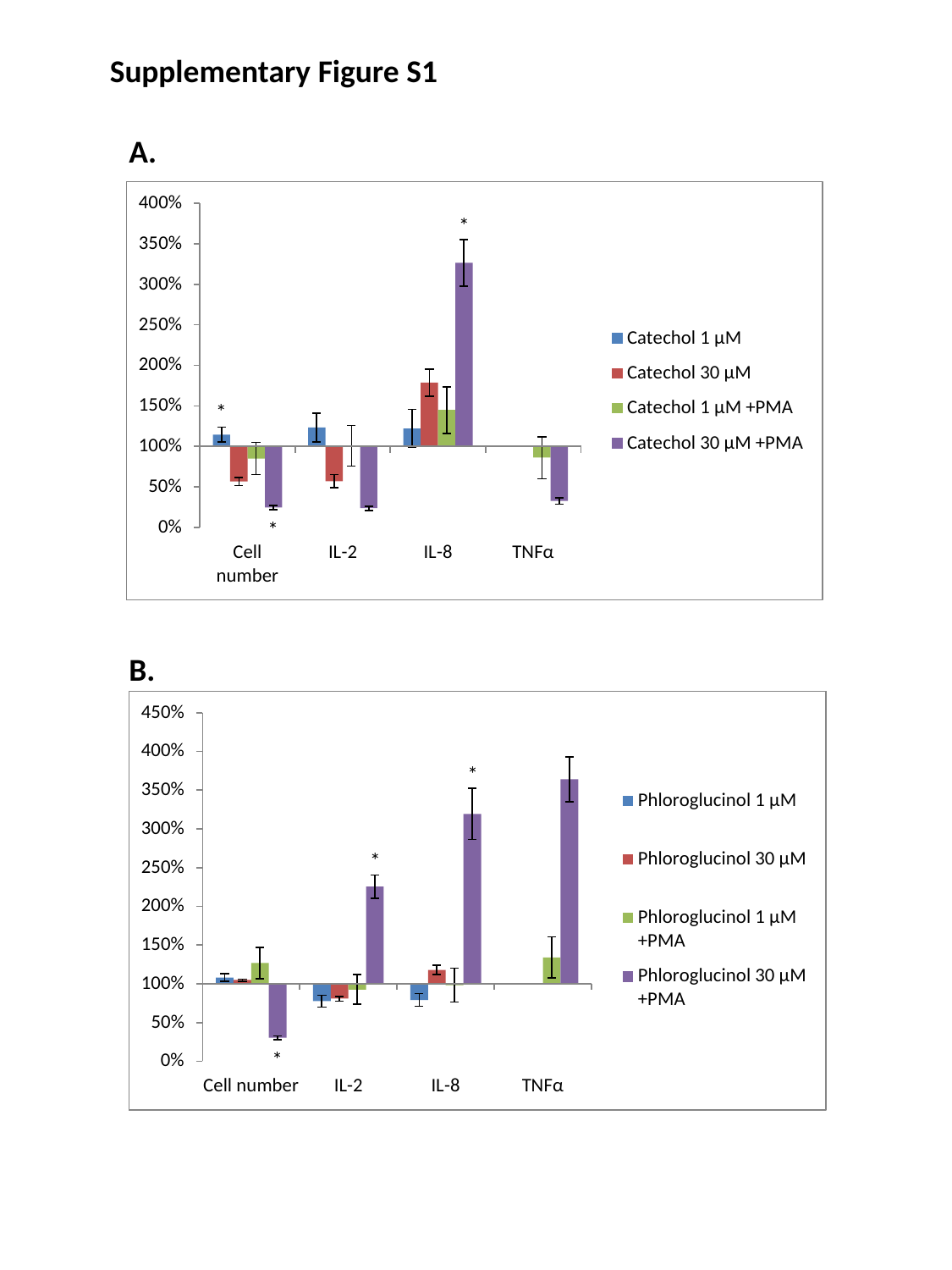

Supplementary Figure S1
A.
B.

## Slide 2
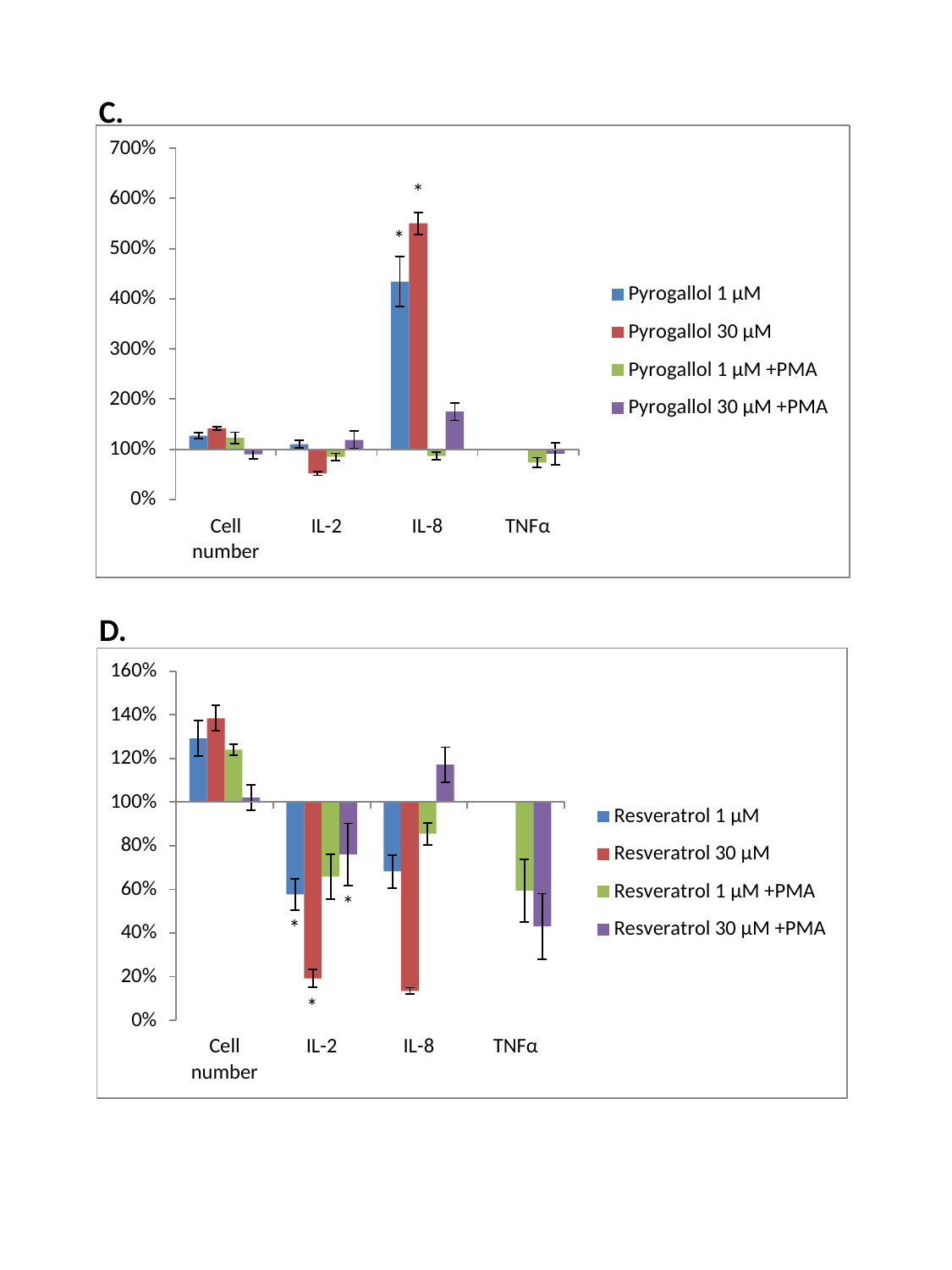

C.
D.

## Slide 3
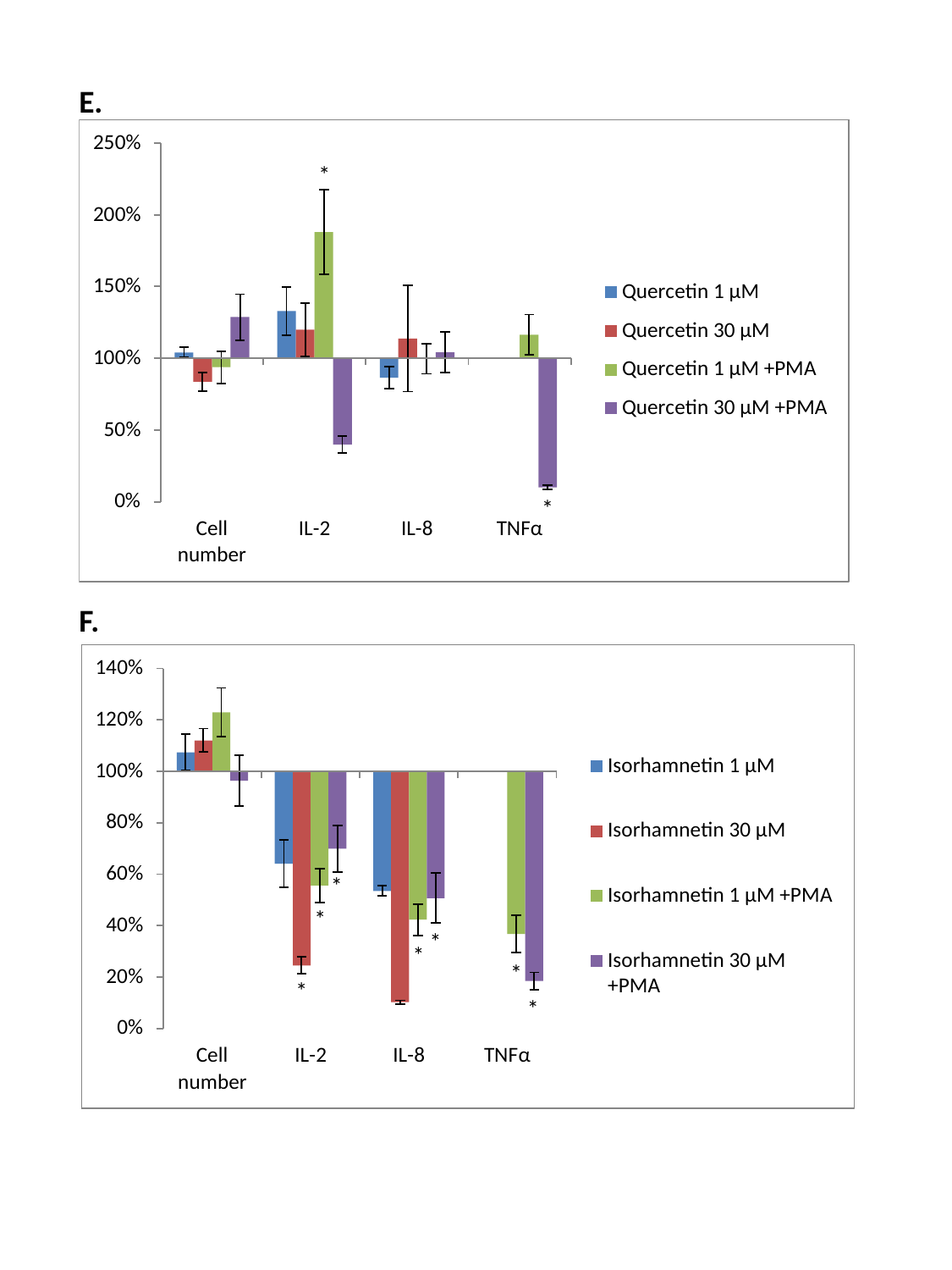

E.
F.

Supplement: Supplementary file 1 [file S0007114516000805sup001.zip › S0007114516000805sup001/S0007114516000805sup001.pptx]
